# The clinical signature of genetic variants and serum levels of macrophage migration inhibitory factor in Egyptian breast cancer patients

**DOI:** 10.1007/s10549-024-07393-9

**Published:** 2024-06-25

**Authors:** Mahmoud A. Seliem, Ahmed M. Mohamadin, Mohamed I. Kotb El-Sayed, Yahia Ismail, Ahmed A. El-Husseiny

**Affiliations:** 1 Biochemistry and Molecular Biology Department, Faculty of Pharmacy, Al-Azhar University, Nasr City, Cairo, 11231 Egypt; 2 Department of Biochemistry, Faculty of Pharmacy, Ahram Canadian University, 6th of October City, Giza, Egypt; 3 Department of Biochemistry and Molecular Biology, Faculty of Pharmacy, Helwan University, Helwan, Cairo, 11790 Egypt; 4 Medical Oncology Department, National Cancer Institute, Cairo University, Cairo, 11796 Egypt; 5 Department of Biochemistry, Faculty of Pharmacy, Egyptian Russian University, Badr City, Cairo, 11829 Egypt

**Keywords:** Breast cancer, Serum MIF, Polymorphism, Immunity, Tumor marker

## Abstract

**Purpose:**

Macrophage migration inhibitory factor (MIF) is an integral cytokine for the modulation of both innate and adaptive immunity and is involved in the pathogenesis of various cancers. However, conflicting findings on the relationship between MIF polymorphisms and breast cancer (BC) have been reported in earlier research. We investigated the clinical value of serum MIF levels and the association between MIF rs1049829 and rs755622 variants with their serum levels and propensity to develop BC.

**Methods:**

A total of 133 treatment-naïve Egyptian BC females and 126 apparently healthy controls were matriculated in this case–control study. The serum MIF protein levels were quantified by ELISA, whereas the genotyping was executed utilizing the TaqMan® allelic discrimination assay.

**Results:**

A significant increase in the serum MIF level in BC cases was observed in comparison to control subjects (*P* < 0.0001), with a diagnostic potential to discriminate BC with 92.5% sensitivity and 73.7% specificity at a cut-off value > 9.47 ng/mL. Besides, a significant difference in serum MIF level was observed in BC cases with progesterone receptor (PR) negativity compared to those with PR positivity (*P* = 0.046). Moreover, a significant association was depicted between the rs1049829 variant of MIF gene and the protective effect against BC meanwhile the rs755622 variant demonstrated no significant link with BC risk.

**Conclusions:**

This study revealed that serum MIF levels may be regarded as a promising serum tumor marker for BC. Also, the rs1049829 variant of the MIF gene is considered a protective candidate against BC.

**Graphical Abstract:**

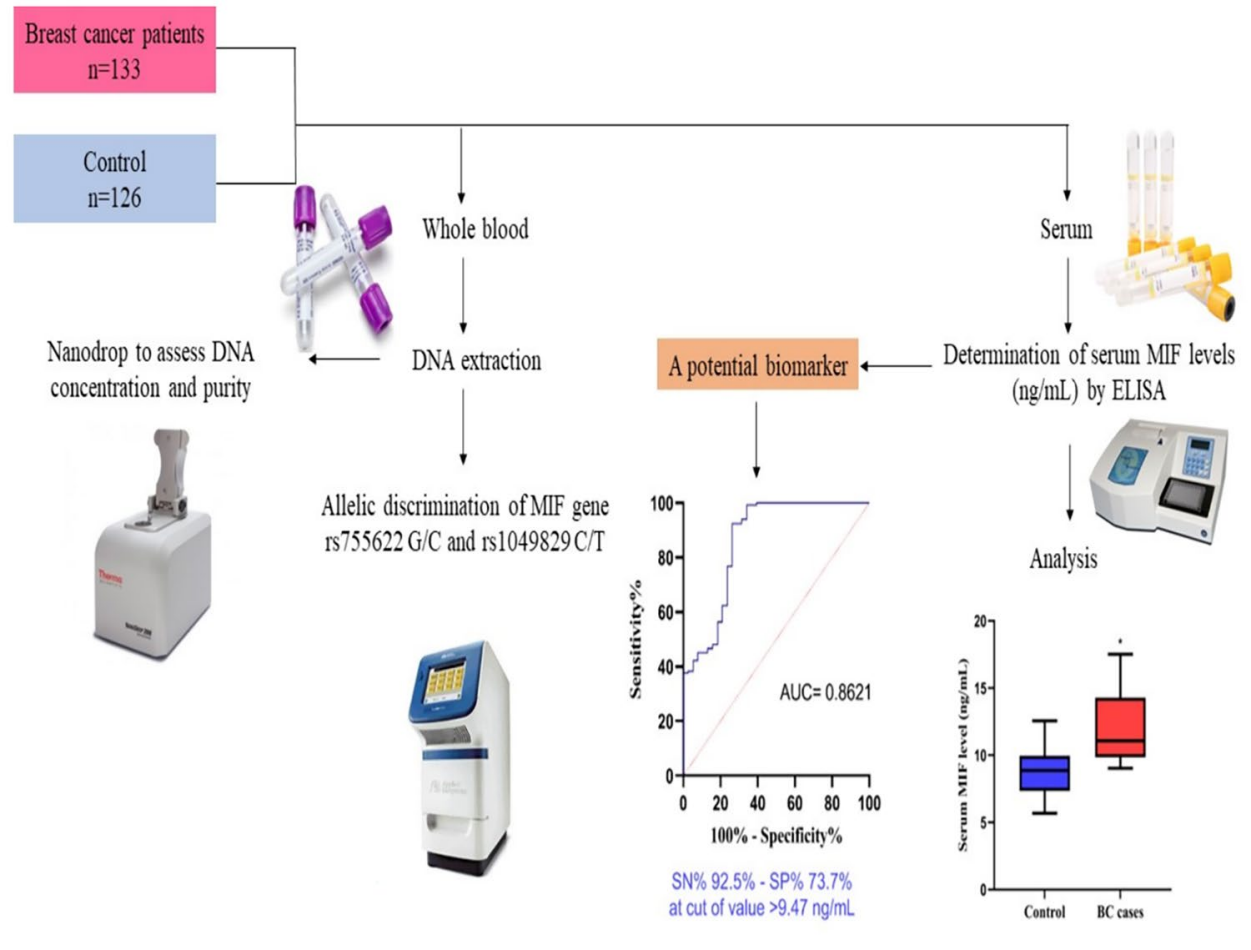

## Introduction

Breast cancer (BC) is the utmost regularly diagnosed malignancy, with an assessed 2.3 million diagnoses and 685,000 fatalities from cancer worldwide in 2020 [1]. The BC mortality rates are considerably increasing in developing countries, including Egypt, which represents 10.8% of total cancer cases [2]. The incidence of BC has noticeably elevated, particularly amongst young females, and its global annual incidence is envisioned to reach 4.4 million in 2070 [3]. Diagnosing BC in women at earlier stages can noticeably enhance the survival rate and the BC prognosis is intensely complicated by late diagnosis [4].

Interestingly, several epidemiological studies revealed that patients enduring chronic inflammatory diseases have an amplified risk for cancer incidence. Besides, it is estimated that about 30% of cancer incidence in low- and middle-income nations is related to microbial infections [5]. With regard to the action of inflammatory mediators in endorsing oncogenesis and tumor progression, evidence is pointing towards a probable correlation amongst macrophage migration inhibitory factor (MIF) expression and tumorigenesis and cancer progression [6].

Macrophages, which have a fundamental function in immune response, were described as a fundamental contributor throughout the oncogenesis process by endorsing tumor cell proliferation, survival, and migration [6, 7]. The MIF gene is located on chromosome 22q11.2 and is considered as a subcomponent of the transforming growth factor-β (TGF-β) superfamily. MIF is released by several cells comprising macrophages, granulocytes, T and B lymphocytes, endothelial cells, as well as cancer cells [8]. The MIF is regarded as a proinflammatory cytokine and contributes in the progress of oncogenesis by endorsing tumor development, altering immunological responses, enhancing inflammation, and aiding cancer-associated angiogenesis [6, 7]. A study by Verjans et al. revealed that MIF protein has a dual role in BC development. Increased MIF protein expression inside the BC cells is beneficial and protective, while increased serum levels of MIF protein are prooncogenic [9].

Some studies reported that the MIF gene rs755622-G/C polymorphism is associated with the amplified solid tumor risk [10] and BC risk [11, 12]. However, the study by Avalos‐Navarro et al. failed to find a notable association in the Mexican‐mestizo population [8]. On the other hand, the rs1049829 variant of MIF gene has been studied for a possible correlation with colorectal cancer risk, but no significant association was found [13]. However, no previous studies were performed to report its association with BC.

There is still no research focusing on the relationship between MIF gene polymorphism and BC susceptibility in Egyptian females. Thus, we investigated the serum MIF levels for the first time in Egyptian BC patients and the association between both MIF gene rs1049829 and rs755622 variants with BC susceptibility.

## Patients and methods

### Subjects

Between December 2022 and March 2023, blood samples were drawn from 133 consecutive treatment-naïve primary BC female patients aged ≥ 18 years who presented to the Medical Oncology Department of the National Cancer Institute of Cairo University, and from 126 healthy females as a control group. Ages were closely matched as feasible between the patient and control groups; the median age of the former group was 50 years (range: 18–65). A definite diagnosis of BC was employed by clinical examination, mammography and confirmed by histopathology. Patients’ demographics and clinicopathological data are presented in Table 1 and Table 2, respectively. Disease stage and tumor grade were based on the American Joint Committee on Cancer (AJCC) [14] and grading approach [15], respectively. We included any disease stage at presentation then patients were divided into two groups: curative stage (I, II, and III) and metastatic stage (IV). According to St. Gallen BC expert consensus [16] and owing to deficient Ki67% information in some BC cases, the remaining patients were classified using immunohistochemistry into classes: luminal A: (ER + and PR + , HER2−, and low-Ki67 index), Luminal B (HER2 negative): (ER + , HER2−, and at least one of high Ki67 or PR-/low), Luminal B (HER2 positive): (ER + , HER2 over-expressed or amplified, and any Ki67 or any PR), triple-negative BC (TNBC): (ER−, PR−, & HER2−), and HER2−enriched (ER−, PR− & HER2+) (Table 2). The existing prospective case–control study was conducted based on the ethical principles of the Helsinki Declaration and was approved by the National Cancer Institute’s Institutional Review Board (IRB), located at Cairo University (Cairo, Egypt), IRB approval no. (2212-5051-0443). In advance, the study protocol was explained to every subject in both written and conversational form. Also, control participants and BC patients signed written informed consents. Exclusion criteria included patients with autoimmune diseases, morbid obesity, tobacco users, or those who received immunomodulators. Healthy female controls were recruited from women whose annual physical examinations revealed no cancer.

**Table 1:** Demographic data of BC cases and controls

Demographic data	BC cases (n = 133)	Controls (n = 126)	*P*
Age at admission	50 (18–65)	49 (21–66)	0.42
Menopausal status			
Premenopausal	68 (51.1%)	71 (56.3%)	0.39
Postmenopausal	65 (48.9%)	55 (43.7%)	
Serum MIF (ng/mL)	11.08 (9.03–17.53)	8.87 (5.67–12.57)	< 0.0001*

BC: breast cancer. Data are expressed as median (range) and n (%)

*Statistically significant at P < 0.05

**Table 2:** Relation between serum MIF level and clinicopathological features of BC patients

Clinicopathologic features		BC patients n (%) (n = 133)	MIF level (ng/mL) median (range)	*P*
Laterality	Right breast	61 (45.8%)	10.79 (9.03–17.53)	0.28
	Left breast	65 (48.9%)	11.85 (9.23–17.52)	
	Bilateral	7 (5.3%)	10.79 (10.060–17.19)	
Primary BC pathology	IDC	114 (85.7%)	11.14 (9.03–17.53)	0.24
	ILC	15 (11.3%)	10.28 (9.23–17.52)	
	Mixed	4 (3%)	13.01 (11.27–15.81)	
pT stage	T1	19 (14.3%)	11.41 (9.48–17.33)	0.69
	T2	55 (41.4%)	11.08 (9.03–17.52)	
	T3	21 (15.7%)	11.79 (9.82–16.83)	
	T4	38 (28.6%)	10.70 (9.23–17.53)	
pN stage	N0	52 (39.1%)	10.63 (9.03–17.52)	0.28
	N1	33 (24.8%)	10.19 (9.28–16.83)	
	N2	27 (20.3%)	11.72 (9.26–17.25)	
	N3	21 (15.8%)	12.76 (9.23–17.53)	
ER status	Negative	31 (23.3%)	11.85 (9.26–17.39)	0.24
	Positive	102 (76.7%)	10.73 (9.03–17.53)	
PR status	Negative	37 (27.8%)	13.02 (9.57–17.39)	0.046*
	Positive	96 (72.2%)	10.63 (9.03–17.53)	
HER2 status	Negative	89 (66.9%)	10.67 (9.03–17.52)	0.06
	Positive	44 (33.1%)	12.03 (9.26–17.53)	
TNM staging	Stage I	11 (8.3%)	13.43 (9.48–16.46)	0.38
	Stage II	44 (33.1%)	10.98 (9.03–17.52)	
	Stage III	39 (29.3%)	10.51 (9.23–17.53)	
	Stage IV	39 (29.3%)	11.70 (9.33–17.25)	
TNM staging groups	Curative (I, II & III)	94 (70.67%)	10.63 (9.03–17.53)	0.23
	Metastatic (IV)	39 (29.33%)	11.70 (9.33–17.25)	
Tumor grading	G1	5 (3.8%)	13.28 (11.41–15.81)	0.19
	G2	95 (71.4%)	10.67 (9.03–17.53)	
	G3	33 (24.8%)	11.72 (9.49–17.33)	
^#^Molecular subtyping	Luminal A	9 (10.6%)	11.89 (9.48–16.65)	0.162
	Luminal B	57 (67.1%)	10.79 (9.23–17.53)	
	HER2-enriched	17 (20%)	13.60 (9.62–17.39)	
	TNBC	2 (2.3%)	15.85 (15.73–15.97)	

*BC* breast cancer, *ER* estrogen receptor, *G* grade, *HER2* human epidermal growth factor receptor 2, *IDC* invasive ductal carcinoma, *ILC* Invasive lobular carcinoma, *pN* pathological nodal, *PR* progesterone receptor, *pT* pathological tumor, *TNBC* triple-negative breast cancer, *TNM* tumor-node-metastasis.

^#^Ki67 data were missed in 48 BC cases. Data are expressed as n (%) or median (range). *Statistically significant at *P* < 0.05.

### Sampling

After the final diagnosis, 6 mL of venous blood specimen was obtained from each subject. 3 mL was collected in a vacutainer tube containing EDTA and stored at −80 °C at Al-Azhar University’s Molecular Biology laboratory until the time of DNA extraction. The other 3 ml was collected in a gel vacutainer tube for serum isolation and used for the determination of serum MIF level.

### Measurement of serum MIF levels

Serum MIF levels were quantified by a sandwich enzyme-linked immunosorbent assay (ELISA) utilizing a commercial human MIF ELISA kit supplied by Elabscience®, Texas, USA (Cat. No: E-EL-H1530) adhering to the producer’s instructions. The optical density was determined utilizing a microplate reader (Stat Fax® 2100, Awareness Technology, USA) set to 450 nm.

### DNA extraction and genotyping

Utilizing the GeneJET™ DNA purification kit (Thermo scientific, USA), genomic DNA was isolated and purified from subjects’ EDTA whole blood. Aliquots of DNA were then kept at −80 °C until analysis. By measuring DNA’s optical density at 260 nm with a Thermo Scientific NanoDrop 2000 spectrophotometer (Wilmington, USA), the concentration of DNA was calculated. At 260/230 nm and 260/280 nm optical density ratios, the purity of DNA was evaluated. Genotyping of MIF single nucleotide polymorphisms (SNPs) rs755622 and rs1049829 was investigated utilizing a TaqMan® allelic discrimination analysis by design provided by Applied Biosystems, Foster City, USA. The manufacturer’s directions and commonly available probes and primers were applied to conduct this investigation employing an Applied Biosystems International Step One real-time PCR apparatus (Foster City, USA) in a volume for each reaction of 20 μL.

### Sample size

Based on Avalos‐Navarro et al. study [8], a sample size of 108 subjects per group was calculated according to the minor allele frequencies of the MIF rs755622 and 1049829 to detect power of 80% and type 1 error = 0.05. Sample size estimation was performed by the PS statistical package.

### Statistical analysis

All statistical assessment was conducted utilizing Graph pad prism (version 9, Inc, USA). The Kolmogorov–Smirnov and Shapiro–Wilk normality tests were applied. The medians and ranges were used to represent quantitative values. On the other hand, numbers (*n*) and percentages (%) were utilized to describe qualitative values. Concerning non-parametric data, the Kruskal–Wallis test was utilized to evaluate the differences across the various studied groups, and if necessary, the Dunn’s adjustment test was performed. Also, the Mann–Whitney U test was applied to evaluate the difference between the two studied groups. Utilizing the receiver operating characteristic (ROC) curve for evaluating the diagnostic accuracy of serum MIF levels, the optimal cut-off point, sensitivity, specificity, as well as area under the curve (AUC) were assessed. Regarding the qualitative data comparison and Hardy–Weinberg equilibrium (HWE) test and genetic association of MIF rs755622 and rs1049829 with risk to BC, the Chi-square test (χ^2^) was employed. For the risk alleles, odds ratios (ORs) and 95% confidence interval (95% CI) were assessed. SHEsis software (http://shesisplus.bio-x.cn/) was applied for haplotype analysis and investigating the interaction of the two MIF SNPs with the BC susceptibility. The criterion for statistical significance was established at *p* < 0.05. Our research adheres to Reporting recommendations for tumor marker prognostic studies (REMARK) standards [17]

## Results

The current study comprised 259 females (133 BC patients and 126 healthy controls). The age distributions of the BC patients and controls were matched. In addition, there was no notable difference in menopausal status between the 2 investigated groups **(**Table 1**)**. Clinicopathologic features of BC cases are summarized in Table 2.

**Fig. 1:**
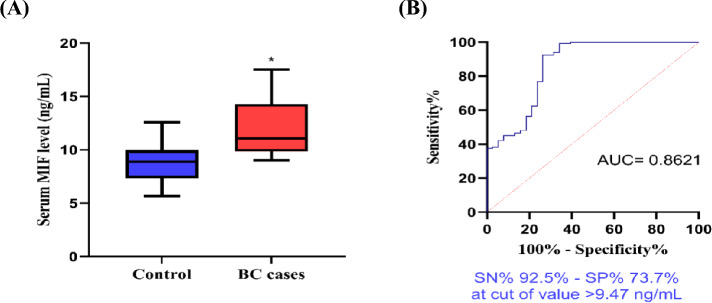
Serum MIF level (ng/mL) in BC cases and controls (**A**) and ROC curve for serum MIF as a biomarker of BC (**B**). *Statistically significant at *P* < 0.05. *AUC* area under the curve, *SN* sensitivity, *SP* specificity

The serum level of MIF was significantly increased in the BC cases in comparison to the control subjects (*p* < 0.0001), with a diagnostic potential to discriminate BC with a sensitivity of 92.5% and a specificity of 73.7% at a cut-off value > 9.47 ng/mL **(**Fig. 1**)**.

**Fig. 2:**
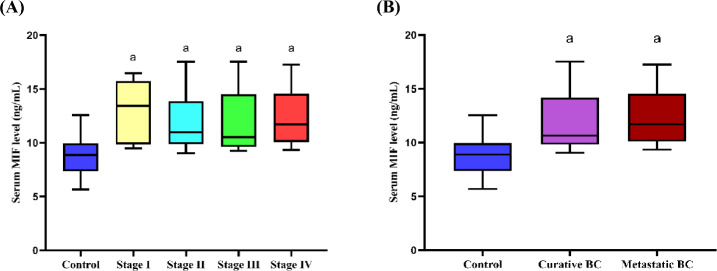
Serum MIF levels (ng/mL) in control subjects and BC cases with different stages (**A**) and those with curative and metastatic stages (**B**). ^a^Significant different from control group at *p* < 0.05

Regarding the comparison of cancer grades and TNM stages in BC patients, there was no notable difference in serum MIF level that was observed. However, a significant increase was demonstrated in BC patients with various TNM stages I, II, III, and IV in contrast to control females with *p* values 0.0002, < 0.0001, < 0.0001, and < 0.0001, respectively **(**Fig. 2A**)**. Also, a significant elevation in serum MIF level was revealed in BC patients with curative and metastatic stages in contrast to control females, with *p* values < 0.0001 and < 0.0001 respectively, while no notable difference in serum MIF levels was demonstrated between BC cases with curative and metastatic stages **(**Fig. 2B**)**.

Owing to the lack of Ki67 data in 48 BC cases, we were unable to classify those patients according to luminal subclassification; however, no marked difference in serum MIF level was observed between BC molecular subtypes in the rest of the cases (*P* = 0.126). Besides, a notable significant difference in serum MIF level in PR-negative compared to PR-positive BC patients (*P* = 0.046), while there was no notable difference observed regarding ER and HER2 expression. Moreover, the statistical findings showed that there was no significant change in serum MIF levels amongst BC patients with various pathological tumor stages (*p* = 0.69) and nodal status (*p* = 0.28) **(**Table 2**)**.

Genotype distributions of the MIF gene rs1049829 and rs755622 variants were in accordance with Hardy–Weinberg equilibrium (*p* > *0.05)* as demonstrated in Tables 3, 4 Interestingly, a significant association between the T allele of rs1049829 variant of MIF gene and the protective effect against BC (OR = 0.51, 95% CI = 0.34–0.77, *p* = 0.001). Besides, a notable association with a protective effect against BC was observed under the two codominant models (CT versus CC, OR = 0.46, 95% CI = 0.27–0.76 *p* = *0.*003, and TT versus CC, OR = 0.24, 95% CI = 0.06–0.85 *p* = *0.*03), the dominant model (OR = 0.43, 95% CI = 0.26–0.72, *p* = 0.001). Moreover, a significant association with the risk of BC was revealed only under the over-dominant model (OR = 1.95, 95% CI = 1.16–3.22, *p* = 0.008), while the recessive model showed no significant association with BC risk (*p* = 0.10) (Table 3).

**Table 3:** Association of rs1049829 variant of MIF gene with a protective effect against breast cancer in the studied subjects

Models	Genotypes and alleles	BC group (n = 133)	Control group (n = 126)	OR	95% CI	*P*
Codominant	CC	85 (63.9%)	55 (43.7%)	1.00 (ref.)		
	CT	45 (33.8%)	63 (50.0%)	0.46	0.27 to 0.76	0.003*
	TT	3 (2.3%)	8 (6.3%)	0.24	0.06 to 0.85	0.03*
Dominant	CC	85 (63.9%)	55 (43.7%)	0.43	0.26 to 0.72	0.001*
	CT+TT	48 (36.1%)	71 (56.3%)			
Recessive	TT	3 (2.3%)	8 (6.3%)	2.93	0.87 to 10.40	0.10
	CC+CT	130 (97.7%)	118 (93.7%)			
Over-dominant	CT	45 (33.8%)	63 (50.0%)	1.95	1.16 to 3.22	0.008*
	CC+TT	88 (66.2%)	63 (50.0%)			
Allelic	C	215 (80.8%)	173 (68.7%)	0.51	0.34 to 0.77	0.001*
	T	51 (19.2%)	79 (31.3%)			
HWE		*P* = 0.57	*P* = 0.19			

*BC* breast cancer, *CI* confidence interval, *HWE* hardy–Weinberg equilibrium, *OR* Odds ratio. *ref.* reference

*Statistically significant different at *p* < 0.05 using Chi-square test.

**Table 4:** Association of rs755622 variant of MIF gene with breast cancer risk in the studied subjects

Models	Genotype and allele	BC group (n = 133)	Control group (n = 126)	OR	95% CI	*P*
Codominant	GG	89 (66.9%)	78 (61.9%)	1.00 (ref.)		
	GC	42 (31.6%)	45 (35.7%)	0.81	0.48 to 1.38	0.45
	CC	2 (1.5%)	3 (2.4%)	0.58	0.10 to 2.93	0.56
Dominant	GG	89 (66.9%)	78 (61.9%)	0.80	0.48 to 1.3	0.4
	GC+CC	44 (33.1%)	48 (38.1%)			
Recessive	CC	2 (1.5%)	3 (2.4%)	1.59	0.32 to 9.10	0.61
	GG+CC	131 (98.5%)	123 (97.6%)			
Over- dominant	GC	42 (31.6%)	45 (35.7%)	1.20	0.71 to 2.03	0.48
	GG+CC	91 (68.4%)	81 (64.3%)			
Allelic	G	220 (82.7%)	201 (79.8%)	0.82	0.53 to 1.27	0.39
	C	46 (17.3%)	51 (20.2%)			
HWE		*P* = 0.48	*P* = 0.49			

*BC* breast cancer, *CI* confidence interval, *HWE* hardy–Weinberg equilibrium, *OR* odds ratio, *ref.* Reference

Statistically significant was established *at P* < 0.05 using Chi-square test.

On the other hand, the statistical data showed no notable association amongst the rs755622 variant of MIF gene and the susceptibility of BC *(p* = 0.39*)*. Moreover, no significant association with the risk of BC was observed under the different genetic models used in this study which include the two codominant (GC versus GG, *p* = 0.45 and CC versus GG, *p* = 0.56), dominant (*p* = 0.4*)*, recessive (*p* = 0.61), and over-dominant (*p* = 0.48*)* (Table 4).

To clarify the association amongst interactions of the two studied MIF SNPs and BC, we have carried out a haplotype analysis performed with in silico analysis by SHEsis software. As a result, the rs755622-G and rs1049829-T haplotypes showed a significant association with a protective impact against BC (OR = 0.51, 95% CI = 0.34–0.79, *p* = 0.002), while other haplotype frequencies showed no significant association with BC risk (Table 5).

**Table 5:** Association between haplotypes of MIF gene and breast cancer susceptibility

Haplotypes		BC group	Control group	OR	95% CI	*P*
rs755622	rs1049829	(n = 133)	(n = 126)			
G	C	175	132	1		
G	T	43	69	0.514	0.335–0.789	0.002*
C	C	38	41	0.861	0.533–1.390	0.54
C	T	8	10	0.772	0.299–1.995	0.59

*BC* Breast cancer, *CI* Confidence interval, *OR* Odds ratio

*Statistically significant different at *p* < 0.05.

**Fig. 3:**
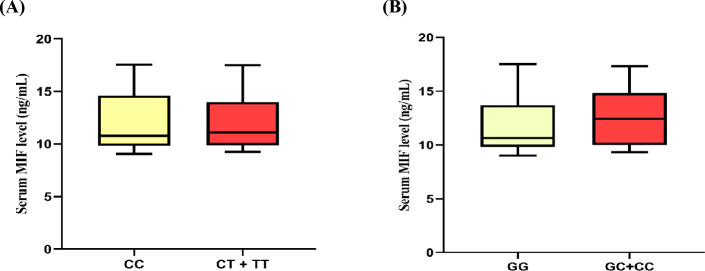
Serum MIF levels in BC patients with different genotypes of rs1049829 C/T (**A**) and rs755622G/C (**B**). Statistically significant was established at *P* < 0.05

In addition, we have investigated the association between serum MIF level and the two investigated MIF SNPs rs755622 and rs1049829 in BC patients, and no statistical association was observed with *p* values 0.1123 and 0.8455, respectively **(**Fig. 3**)**.

## Discussion

Breast cancer is the foremost determinant of cancer mortality in females [1]. Successful cancer treatment requires the development of early-stage cancer diagnostic techniques and the assessment of a patient’s risk of cancer progression and recurrence [18]. Several studies have informed that the existence of genetic factors, such as mutations and SNPs, increases the risk for the development and progression of BC [19], as well as those found in the proinflammatory cytokine MIF gene [11]. The contribution of the MIF alleles in the progression of cancer was correlated and reported in different types of cancers such as hepatocellular carcinoma [20], gastric cancer [21], BC [11], and prostate cancer [22]. This is the first study, to our knowledge, to assess the allelic and genotypic frequencies of (rs1049829) and (rs755622) polymorphisms of MIF gene in women with BC in the Egyptian population.

To the best of our knowledge, the association between the MIF gene rs1049829 and BC risk has not been previously studied. This study describes a significant correlation between the rs1049829 variant of the MIF gene and the protective effect against BC in women from the Egyptian population. Although a study by Ramireddy et al. found no notable correlation amongst this polymorphism and the susceptibility to colorectal cancer in the Taiwan population [13]. This discrepancy may be referred to differences in cancer type, sample size, age, and race.

Consistent with our findings, a study by Avalos‐Navarro et al. evaluated the MIF gene rs755622-G/C polymorphism in women with BC in the Mexican population and showed no significant correlation with BC risk [8]. Conversely, a study by Lin et al. showed a notable association between the rs755622 variant of the MIF gene and BC susceptibility in women of the Chinese population [11]. Further studies found a protective effect for this SNP against colorectal cancer [23] and bladder cancer risks [24, 25]. These disagreements may be credited to the criteria for inclusion, sample size, and racial variances amongst nations. Also, some findings from earlier research should be interpreted cautiously because not all genetic models have been evaluated in these investigations.

In accordance with other reports [8, 26, 27], the serum MIF level in BC patients is markedly elevated than that in control subjects, suggesting that MIF has a marked role in endorsing oncogenesis. Furthermore, we demonstrated that serum MIF has the diagnostic potential to distinguish between BC patients and healthy controls at a cut-off value of > 9.47 ng/mL with a 92.5% sensitivity and 73.7% specificity. In harmony with our results, Ciftci et al., observed a significant difference in serum MIF level between BC cases and control group, with a mean level of 10.7 and 5.49 ng/mL, respectively. Also, they revealed a discriminating power of serum MIF level between BC patients and healthy individuals at a cut-off value of 1.1275 ng/ml with sensitivity and specificity of 97% and 71%, respectively [27]. The difference between our observed cut-off value and that of Ciftci et al. may be attributed to the difference in age of recruited subjects and sample size where Ciftci et al. recruited only 28 control subjects and 96 BC cases. Besides, Fersching et al. revealed a discriminating ability of MIF between BC patients and healthy individuals, and between metastatic BC patients and BC patients with locally confined, with AUC 70.7% and 87.6%, respectively [28]. Similarly, MIF was found to be a possible diagnostic marker for gastric cancer at a cut-off value of 3.23 ng/mL with 83.5% sensitivity, 92.3% specificity, and 89.7% accuracy [29]. While the difference observed in cut-off value may be attributed to the difference in cancer type, gender recruitment, and patient demographics. Moreover, Richard et al. revealed a marked increase in the expression level of MIF protein inside the BC tissue describing MIF as a marker to discriminate normal tissue from BC tissue by immunohistochemistry [30]. These findings might enhance the potentiality of serum MIF level as a diagnostic tumor marker for BC, point out MIF as a possible therapeutic target for pharmacological modulators, and indicate the evidence that cytokines are spawned by immune and tumor cells.

Interestingly, the primary source of MIF in tumors is the epithelial cells themselves, with a little secretory supply from stromal and inflammation-related cells, as well as other components of the tumor microenvironment. Notably, the cytokine MIF drives oncogenesis by supporting tumor growth, regulating immunological responses, boosting inflammation, and promoting tumor-associated angiogenesis [31, 32]. Also, MIF enhances the cancerous microenvironment by inducing inflammation and releasing inflammatory mediators like tumor necrosis factor α, interleukin (IL)-1β, and IL-6 [33].

The present study hasn’t found a statistical significance in serum MIF levels between different grades and stages of BC. These results are endorsed by several studies that reported that neither MIF expression in BC tissue [30, 34] nor serum MIF level [30] showed statistically significant differences between different tumor grades. However, results obtained by Avalos-Navarro et al. revealed a statistically significant elevation in serum MIF levels in BC cases with advanced stages compared to those with stage I [35]. This contrasting finding may be attributed to the difference in the sample size, patients’ inclusion criteria, and the utilized kit.

Furthermore, we depicted that elevated serum MIF levels were associated with PR negativity, this result comes in harmony with other reports that showed that the expression of the MIF gene is elevated in cells with negative hormonal receptors [9, 35, 36]. Such finding might signify that MIF is a probable marker for aggressive BC conditions characterized by poor prognosis, recurrence, metastasis, and lack of specific therapeutic targets [37], suggesting a potential use of anti-MIF agents, such as Imalumab which is still processed in clinical trials and showing promising antitumor activity in patients with advanced solid tumors. Imalumab increases apoptosis by suppressing MIF-induced phosphorylation of ERK1/2 and AKT. Imalumab also makes cancer cell lines more susceptible to cytotoxic medications [38]. Moreover, in clinical practice, serum MIF levels could be utilized to stratify patients based on the aggressiveness of their disease, potentially guiding treatment decisions. This stratification could inform the choice and intensity of therapeutic interventions.

In addition, we have evaluated the probable relationship amongst the functional variants of the MIF gene (rs755622 and rs1049829) with the serum levels of MIF protein in women with BC and showed no significant variation in serum MIF levels neither the BC patients with rs1049829 TT and CT versus CC nor rs755622 CC and GC versus GG. There are limitations in this study, Ki67 data were lacking for some BC patients which decreased the number of cases in the molecular subtyping; moreover, this case–control study did not clarify the underlying mechanisms beyond the effect of MIF polymorphisms on BC risk. Therefore, laboratory validation both in vivo and in vitro is envisioned to illuminate the detailed molecular mechanism, and prospective clinical validation with studies involving larger cohorts of breast cancer patients is required to confirm the prognostic value of serum MIF levels. These studies should aim to correlate serum MIF levels with clinical outcomes, including response to treatment and overall survival.

## Conclusions

The findings of this research proposed that serum MIF level may be considered a helpful tumor marker of BC. Also, the rs1049829 variant of MIF gene is considered a protective candidate against BC whereas the rs755622 variant is not regarded as a genetic risk factor for BC amongst Egyptian patients. Moreover, the MIF gene haplotype (rs1049829-T and rs755622-G) showed a significant association with a protective effect against BC.

## Data Availability

The datasets generated during and/or analyzed during the current study are available from the corresponding author on reasonable request.

## Abbreviations

AJCC: American joint committee on cancer
AUC: Area under the curve
BC: Breast cancer
ELISA: Enzyme-linked immunosorbent assay
ER: Estrogen receptor
G: Grade
HER2: Human epidermal growth factor receptor 2
HWE: Hardy–Weinberg equilibrium
IRB: Institutional Review Board
IDC: Invasive ductal carcinoma;
ILC: Invasive lobular carcinoma
MIF: Macrophage migration inhibitory factor
pN: Pathological node
PR: Progesterone receptor
pT: Pathological tumor
ROC: Receiver operating characteristic
SNPs: Single nucleotide polymorphisms
TGF-β: Transforming growth factor-β
TNBC: Triple negative breast cancer
TNFα: Tumor necrosis factor alpha
TNM: Tumor-node-metastasis
